# 

**Published:** 1852-07

**Authors:** 


Philadelphia.

---

### CONTENTS

OF THE

### MEDICAL EXAMINER.

NEW SERIES—NO. XCI—JULY, 1852.

### ORIGINAL COMMUNICATIONS.

On the Functions of the Great Sympathetic Nerve, or the Nerve of Organic Life. By William Gries, M. D., of the city of Reading, Pennsylvania, - - - - - - 413

Remarks on Medical Organization. By Henry Hartshorne, M. D., of Philadelphia, - - - - - - 420

On Chloroform as an Emmenagogue. By David H. Gibson, M. D., Fort Towson, Choctaw Nation, - - - - 425

A New Instrument for Cauterizing the Urethra. By E. S. Cooper, M. D. Reported by L. C. Lane, M. D., of Peoria, Ill., - 427

On the Nutrition of Muscles during their Contraction. By E. Brown-Séquard, M. D., of Paris, - - - - - 428

### CLINICAL REPORTS.

Clinic of Jefferson Medical College. Service of Professor Dunglison. Feb. 21st, 1852. Reported by J. Aitken Meigs, M. D., - 430

### BIBLIOGRAPHICAL NOTICES.

Elements of Chemistry, including thé Applications of the Science in the Arts. By Thomas Graham, F. R. S., Professor of Chemistry in University College, London, &c. &c. Second American, from an entirely revised and greatly enlarged English Edition; with numerous wood engravings. Edited, with notes, by Robert Bridges, M. D., Professor of Chemistry in the Philadelphia College of Pharmacy, &c., &c., - - - - 435

A System of Operative Surgery, based upon the Practice of Surgeons in the United States, and comprising a Bibliographical Index and Historical Record of many of their Operations for a period of two hundred years. By Henry H. Smith, M. D., &c. &c., 438

Obstetrics: the Science and the Art. By Charles D. Meigs, M. D., Professor of Midwifery and Diseases of Women and Children in Jefferson Medical College, at Philadelphia, &c. &c., - 441

The Medical Student's Vade Mecum; a Compendium of Anatomy, Physiology, Chemistry, Materia Medica and Pharmacy, Surgery, Obstetrics, Practice of Medicine, Diseases of the Skin, Poisons, &c. &c. By George Mendenhall, M. D., - - 442

### EDITORIAL.

Explanation, - - - - - - - - 443

Resolutions relative to the Resignation of Dr. John Bell, - - 443

### MEDICAL NEWS.

Extract from an Address delivered by Dr. Samuel Grimes, of Delphi, at New Albany, May 20th, 1852, before the Indiana Medical Convention, - - - - - - - - 445

Present to the Royal College of Surgeons, - - - - 449

Opposition to Vaccination, - - - - - - 449

The Concours for the Chair of Hygiene, in the Faculty of Medicine of Paris, - - - - - - - 449

Progress of Epidemics, - - - - - - 449

Death of Dr. John Dalrymple, - - - - - 450

M. Chomel, - - - - - - - - 450

### RECORD OF MEDICAL SCIENCE.

### MATERIA MEDICA AND THERAPEUTICS.

On the Basis of the Eau Medicinale D'Husson. By Thomas Bushell, Esq., - - - - - - - 450

### PATHOLOGY AND PRACTICE OF MEDICINE.

St. Bartholomew's Hospital.—Aneurism by Anastomosis; Death by Chloroform. (Under the care of Mr. Lloyd,) - . 454
The Mechanism of Bronchophony. By W. H. Walshe, M. D., Professor of the Principles and Practice of Medicine, University College, London, &c., - - - - - - 456
London Hospital.—Abscess of the Lung following Pneumonia; Death; Autopsy. (Under the care of Dr. Pereira,) - - - 463
A Case of Hydrophobia in the South of France, - - - 464
Remarks on the Microscopic Characters of the Urine in Bright's Disease of the Kidney. By John D. Macdonald, Esq., R. N., - 466

### SURGERY.

Charing Cross Hospital.— Convulsive Movements of Stump; Removal of the Nervous Bulbs; Amputation at the Shoulder-joint; Persistence of the Spasmodic Jerking. Under the care of Mr. Hancock, - - - - - - - 468

### OBSTETRICS.

Parturition among the Esquimaux, - - - - - 473
On the meaning of the term "Ulceration," as at present applied to Uterine Diseases. By T. Snow Beck, M.D., F.R.S., Physician to the Farrington General Dispensary and Lying-in Charity, - 474

### ANATOMY AND PHYSIOLOGY.

On the Occasional Organic Union of Contiguous Teeth. By S. J. A. Salter, M. B., etc., - - - - - - 479
On the Molecular Organ of the Tissues. By Dr. Bennett, - - 480

